# Deep LSTM-Based Transfer Learning Approach for Coherent Forecasts in Hierarchical Time Series

**DOI:** 10.3390/s21134379

**Published:** 2021-06-26

**Authors:** Alaa Sagheer, Hala Hamdoun, Hassan Youness

**Affiliations:** 1 College of Computer Sciences and Information Technology, King Faisal University, Al-Ahsa 31982, Saudi Arabia; 2 Center for Artificial Intelligence and Robotics (CAIRO), Aswan University, Aswan 81582, Egypt; hala@aswu.edu.eg; 3 Department of Computers and Systems Engineering, Faculty of Engineering, Minia University, Al-Minia 61519, Egypt; hassan_youness@mu.edu.eg

**Keywords:** deep long short-term memory, auto-encoder, hierarchical time series, coherent forecast, power generation, australian tourism

## Abstract

Hierarchical time series is a set of data sequences organized by aggregation constraints to represent many real-world applications in research and the industry. Forecasting of hierarchical time series is a challenging and time-consuming problem owing to ensuring the forecasting consistency among the hierarchy levels based on their dimensional features. The excellent empirical performance of our Deep Long Short-Term Memory (DLSTM) approach on various forecasting tasks motivated us to extend it to solve the forecasting problem through hierarchical architectures. Toward this target, we develop the DLSTM model in auto-encoder (AE) fashion and take full advantage of the hierarchical architecture for better time series forecasting. DLSTM-AE works as an alternative approach to traditional and machine learning approaches that have been used to manipulate hierarchical forecasting. However, training a DLSTM in hierarchical architectures requires updating the weight vectors for each LSTM cell, which is time-consuming and requires a large amount of data through several dimensions. Transfer learning can mitigate this problem by training first the time series at the bottom level of the hierarchy using the proposed DLSTM-AE approach. Then, we transfer the learned features to perform synchronous training for the time series of the upper levels of the hierarchy. To demonstrate the efficiency of the proposed approach, we compare its performance with existing approaches using two case studies related to the energy and tourism domains. An evaluation of all approaches was based on two criteria, namely, the forecasting accuracy and the ability to produce coherent forecasts through through the hierarchy. In both case studies, the proposed approach attained the highest accuracy results among all counterparts and produced more coherent forecasts.

## 1. Introduction

Forecasting is one of the pillars for decision support systems in different domains such as weather, web traffic, demand and sales, and energy. It can be defined simply as the rational prediction of future events based on past and current events, where all events are represented as time series observations [1]. Time series forecasting (TSF) with reasonable accuracy is a necessary but quite tricky problem because it correlates many factors with an enormous amount of observations [2].

Many real-world applications exhibit naturally hierarchical data organization, with multiple time series on several levels based on dimensional attributes such as geography, products, or some other attributes. Forecasting through such a hierarchical time series (HTS) structure adds difficulty to the original TSF problem owing to ensuring the forecasting consistency among the hierarchy levels, a phenomenon called coherency [3]. Coherent forecasts mean that the forecast of a time series in a higher level should equal to the sum of forecasts of the corresponding time series in the lower level. In other words, to ensure forecast coherency, we require the forecasts to add up in a manner that is consistent with the aggregation structure of the collection of time series [4]. Therefore, it is often necessary to carry out a reconciliation step to adjust the lower level forecasts and to make them coherent or consistent in the upper levels [5].

In the past two decades, several reconciliation procedures are presented to ensure coherent forecasts, including top-down, bottom-up, and a combination of both procedures called middle-out [6]. These procedures generate what we can call “base” forecasts in different ways by separately predicting individual time series. Then, the “base” forecasts are reconciled according to the inherent hierarchical structure of the selected procedure. The bottom-up procedure starts by estimating the “base” forecasts for the time series at the bottom level in the hierarchy. Then, it implements a simple aggregation way to obtain the “target” forecasts at the higher levels of the hierarchy. On the contrary, the top-down procedure involves estimating the “base” forecasts only for the top layer of the hierarchy and then disaggregates it based on historical proportions of time series at the lower levels. The middle-out procedure estimates the “base” forecasts for the time series at the intermediate level and then implements both bottom-up and top-down procedures to perform the prediction through the hierarchy [6].

Overall, it is not ultimately confirmed which procedure is more efficient than the other because every procedure focuses on a different aggregation level to yield forecasts [7]. Researchers have attributed such instability to the tendency of these procedures to minimize the forecast errors without questioning the coherence of consecutive predictions. As a result, some important information that exists in other levels will be ignored [8]. If we disregard the aggregation constraints, we could forecast all the time series in a group independently. Nevertheless, it is still doubtful that the produced set of forecasts is coherent [5]. Another drawback in the existing approaches is that none of them takes account of the relationships and inherent correlation among series. Therefore, it is not easy to identify prediction intervals for the forecasts from any of these approaches [6].

Toward optimal reconciliation, Hyndman et al. introduced an optimal combination approach that outperformed the approaches mentioned above [6]. This approach assumes that the forecasting error distribution is typical as the aggregated hierarchical structure [9]. It runs by estimating forecasts on all levels of the hierarchy independently. Then, it uses a regression model to optimally combine these forecasts to yield a set of coherent forecasts [6,9]. The generated forecasts add up appropriately across the hierarchy, are unbiased and have minimum variance amongst all combination forecasts [3]. However, this approach is computationally expensive, particularly for large hierarchies, because it should estimate forecasts for all levels in the hierarchy [10,11]. In addition, the authors did not provide any justification whether the reconciled forecasts work well compared to the most commonly implemented approaches [5].

As a common practice in all the approaches that mentioned above, the “base” forecasts are generated using either a statistical or empirical forecasting method depending on the aggregation level of focus [12]. Standard statistical methods include autoregression integrated moving average (ARIMA), Box and Jenkins method, and exponential smoothing (ES) method [13]. These statistical methods are static and often ignore the dynamics of the individual time series and the structure of grouped time series during computations. Consequently, they may fail to perform well if the corresponding groups of time series are subject to a time variation or any sudden change, as the case in energy load or supply chain applications [14]. In addition, it was demonstrated that these methods failed to exploit the complete available information in the hierarchy, which influences the efficiency of the overall forecasting. Moreover, they lack a straightforward mean to determine an optimal reconciliation approach, which is crucial for hierarchical aggregation [9].

Different machine learning algorithms were recently utilized to estimate the “base” and “target” forecasts and to derive the combination weights for the forecasts in the optimal combination approach. For example, Mahdi et al. [14] used artificial neural networks (ANN), extreme gradient boosting (XGboost), and support vector regression (SVR) algorithms to estimate the proportions of time series through a middle-out approach. Spiliotis et al. [12] suggested a machine learning-based method that allows for a nonlinear combination of the “base” forecasts more general than other linear methods. It estimates the “base” forecasts directly without conditioning that the complete information is utilized to generate reconciled forecasts for each time series. Shiratori et al. [15] introduced an approach based on a forecasting model for each time series at the bottom level and then used a structured regularization step to combine the forecasts at the upper levels into the forecast of the bottom level. Mancuso et al. [16] represented the disaggregation method as a nonlinear regression procedure instead of relying on forecast or historical proportions. Namely, they proposed a deep neural network (DNN) model that learns how to share the top level forecasts to the time series at the bottom level of the hierarchy, taking into consideration the characteristics of the information of the individual series and the aggregated series.

To the best of our knowledge, some machine learning-based reconciliation approaches adopted the top-down approach and exploited information only from the parent node. They ignore the rest of the nodes that could be beneficial for obtaining more accurate results, such as the system shown in [14]. Some other machine learning-based approaches try to optimize the forecast accuracy in a linear coherence fashion, such as the system shown in [5], which adopted an in-sample error method for the baseline forecasting. This approach may not be representative of out-sample accuracy [12]. Overall, most machine learning-based reconciliation approaches are built to achieve coherency under particular assumptions to improve the overall prediction accuracy. An exception is the approach presented in [12], which solved the problem of forecasts reconciliation in a general nonlinear fashion to enhance the overall performance across all levels of the hierarchy. However, this approach has a reservation of bias since it selects the forecasts to be combined and neglect other forecasts that may be useful. Nevertheless, all of these approaches still use traditional methods, such as ARIMA and ES, to generate the “base” forecast.

In the last two years, the first author of this paper developed a Deep Long Short-Term Memory (DLSTM) approach that can solve various forecasting problems using either univariate or multivariate time series data [17,18]. The excellent empirical performance of DLSTM motivated us to extend it to treat the hierarchical time series forecasting (HTSF) problem. In this paper, we develop the DLSTM in an auto-encoder (AE) fashion; from here, on we call it DSLTM-AE, taking full advantage of the hierarchical architecture for better time series forecasting. DLSTM-AE can work as an alternative approach to traditional and machine learning approaches that have been used to manipulate the HTSF problem. However, as demonstrated in [18], training a DLSTM in hierarchical architectures requires updating the weight vectors for every LSTM cell, which is time-consuming and requires a large amount of data through several dimensions. Transfer learning strategy can mitigate the effect of this problem, particularly if we adopt it in a bottom-up procedure. The proposed approach runs as follows: First, we train the time series at the bottom level of the hierarchy using DLSTM-AE and generate the “base” forecasts. Then, we use (or transfer) the learned features of the “base” forecast to perform synchronous training to all time series at the upper levels of the hierarchy to estimate the “target” forecasts.

To demonstrate the efficiency of the proposed approach, we empirically compared its performance with several existing approaches using two different case studies using two different datasets. Namely, the Brazilian electrical power generation dataset [19] and the Australian visitor nights of domestic tourism dataset [20]. Using three different performance metrics, we compared our results with those reported by the authors of [19,20], respectively. The comparison among all approaches was based on two criteria: forecasting accuracy and the ability to produce coherent forecasts. Overall, the proposed approach attained the highest accuracy results among all reference approaches. Moreover, it produced more coherent forecasts through a straightforward scenario compared to reference approaches.

The DLSTM-AE procedure and its empirical results shown in the following sections highlight additional advantages of this approach. First, the DLSTM-AE utilizes all the information included in all time series in all levels of the hierarchy, contrary to reference approaches that may ignore useful information. Indeed, this is expected to enhance the overall forecasting performance of the approach. Second, the DLSTM-AE produces forecasts at each level of the hierarchy, which provides meaningful insights for decision-makers to make decisions at any moment during the operation, such as demand planning, production planning, energy crisis, etc. Third and in terms of the original LSTM properties, the DLSTM-AE inherently considers the relationship and temporal correlations among time series observations and automatically extracts many inherent useful features [21]. Fourth, as the computations at the higher levels are performed in synchronous mode, this undoubtedly reduces the whole process’s computational cost.

Consequently, we can summarize our contribution in this paper as follows:

1. We developed a novel deep neural network model runs in a transfer learning scenario that significantly simplifies the forecasting through hierarchical architectures.
2. Our approach attained the highest forecasting accuracies among existing approaches.
3. Our approach produced more coherent forecasts within the hierarchy levels using a straightforward procedure.
4. We made all source codes and a standalone implementation available on GitHub https://github.com/Hala-Hamdoun/DLSTM-AE.git accessed on 25 June 2021.

The rest of the paper is organized as follows. Section 2 briefly summarizes the standard time series forecasting problem and its related concepts. The elements and steps of the proposed approach are presented in Section 3. Section 4 shows the experimental settings and datasets used in this paper. The empirical results achieved using two case studies and related analysis are provided in Section 5. Finally, an overall discussion and the paper conclusions are presented in Section 6.

## 2. Time Series Forecasting

A Time Series Forecasting (TSF) system involves predicting the system performance in the future based on information from the current and past status of the system. It works by considering a sequence of observations (*x*) measured in proper chronological order of a variable of interest at time (*t*). A sequence of time series data can be described as:

(1) $$X=(x_{t};\;t=1,\ldots ,N)$$

where *X* is the time series sequence and *t* refers to the observation’s time for *N* observations. It is worth mentioning that the TSF problem is not limited to forecasting the observation of the next time step only, which is called single-step ahead forecast. There are also time series problems that involve forecasting a sequence of future values, which is known as multi-step ahead forecast [22].

### 2.1. Single-Step ahead Forecast (SSaF)

It is the simplest method in time series forecasting as it just forecasts only the next future value. Simply, SSaF at a time *t* + 1 is performed by passing the current and the past observations *t*, *t* − 1, *t* − 2,…, *t* − *N* to a selected model [23],

(2) F(t+1)=M(o(t),o(t−1),o(t−2),…,o(t−N))

where F(t+1) is the forecast at time (t+1), *M* is the proper selected model, and o(t) is an observation at time *t*.

### 2.2. Multi-Step ahead Forecast (MSaF)

MSaF involves predicting the next *H* sequence of future time series values $\left[y_{1},y_{2},\ldots ,y_{N}\right]$ with *N* observations and with *H* > 1 being prediction horizon. The three main MSaF strategies adopted in the literature are recursive strategy, direct strategy, and multi-input multi-output strategy (MIMO) [22]. In the following, we describe these three strategies, where *f* and *F* denote the functional dependency between past and future observations, and *d* represents the number of past values used to forecast the future values.

1. Recursive strategy (also known as iterated or multi-stage) is the oldest and most intuitive strategy used to treat MSaF [24,25]. In this strategy, a single model *f* is trained to perform only a one-step ahead forecast. Then, the value that is just forecasted is used as part of the following input variable to forecast the next step with the same step ahead model. Accordingly, all forecasts for the entire horizon *H* are obtained as described in the above manner. Considering the trained one-step ahead model $\hat{f}$, the forecasts are given as follows:
  (3) $${\hat{y}}_{N+h}=\left\{\begin{array}{cc} \hat{f}\left(y_{N},\ldots ,y_{N-d+1}\right), & \mathrm{if}h=1\\ \hat{f}\left({\hat{y}}_{N+h-1},\ldots ,{\hat{y}}_{N+1},y_{N},\ldots ,y_{N-d+1}\right), & \mathrm{if}h\in 2,\ldots ,d\\ \hat{f}\left({\hat{y}}_{N+h-1},\ldots ,{\hat{y}}_{N-d+h}\right), & \mathrm{if}h\in d+1,\ldots ,H \end{array}\right.$$
  It is worth mentioning that the recursive strategy showed low performance in some multi-step ahead forecasting tasks due to the noise present in the time series and the forecasting horizon. In such a case, the produced prediction error propagates forward as long as the forecasting horizon increases. These forecasts are used as an input value for making subsequent forecasts [22].
2. Direct strategy (also known as independent strategy) depends on predicting each horizon independently from the others [24,25]. The forecasts are given as follows:
  (4) $${\hat{y}}_{N+h}={\hat{f}}_{h}\left(y_{N},\ldots ,\ldots ,y_{N-d+1}\right)$$
  The direct strategy does not use any approximated values to compute the forecasts, protecting them from the error accumulation formed by the recursive strategy. However, the *H* models are learned independently, inducing conditional independence of the *H* forecasts. This resulted in a negative effect on the forecasting accuracy. On the other hand, it is known that this strategy does not consider complex dependencies between the variables [22]. Therefore, it is an expensive computational strategy considering that it requires many models as the size of the adopted horizon [22].
3. Multi-input multi-output (MIMO) strategy learns the dependency between past observations and future values directly from data. In this case, the future values are a vector of values of a time series itself instead of scalar quantities [26,27]. The merits of the MIMO strategy is its capability to preserve the temporal stochastic dependency included in the predicted future values as it uses only one multi-output model. It constrains all the horizons to be predicted with a unified structure using the same learning algorithm. As a result, the forecasts are obtained in one-step at once by adopting the following multi-output model $\hat{F}$:
  (5) $$\left[{\hat{y}}_{t+H},\ldots ,{\hat{y}}_{t+1}\right]=\hat{F}\left(y_{N},\ldots ,y_{N-d+1}\right)$$

Regardless of the advantages and disadvantages of the first two strategies, it is clear that their original ideas still run as single-output functions but with multiple inputs. Indeed, prediction using a single-output function ignores the presence of stochastic dependencies among future values, which influence the overall prediction performance [28]. In contrast, many real-world applications have adopted this strategy to model and analyze univariate and multivariate TSF problems. In this paper, we extend the MIMO strategy to treat the HTSF problem summarized in the following subsection.

### 2.3. Hierarchical Time Series Forecasting

**Figure 1:**
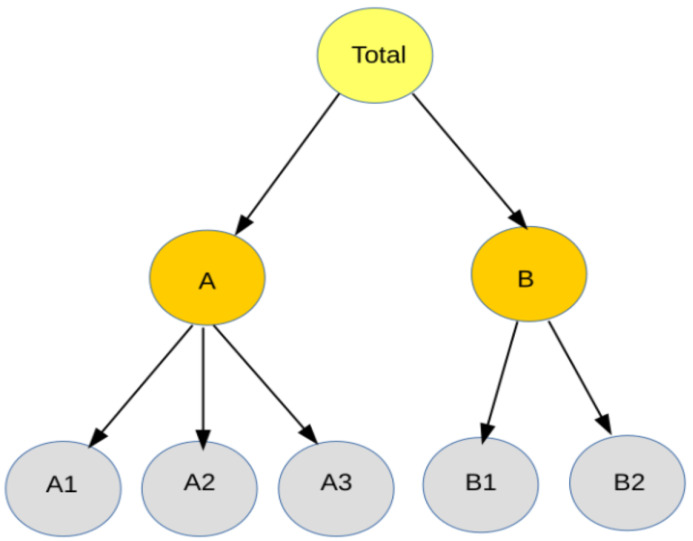
Two-level hierarchical time series structure.

A hierarchical time series (HTS) is a collection of time series that follows a hierarchical aggregation structure with more than one level. Figure 1 demonstrates a simplified hierarchy, but a general hierarchical structure has K>0 levels, where level (0) contains the complete aggregated (top) time series. Each level from (1) to (K−2) denotes a further disaggregation down to the level (K−1), which contains the most disaggregated (bottom) time series [5]. The structure of the hierarchy is determined by the summing matrix *S* that defines the aggregation constraints:

(6) $$y_{t}=Sy_{K,t}$$

where $y_{t}$ is a vector of all observations in the hierarchy at time *t*; *S* is the summing matrix of order *m* × $m_{K-1}$ that aggregates the bottom level series; and *m* and $m_{K-1}$ denote the total number of series in the hierarchy and the number of series at the bottom level, respectively.

The hierarchy shown in Figure 1 can be expressed as follows:

(7) $$\left[\begin{array}{c} y_{t}\\ y_{A,t}\\ y_{B,t}\\ y_{A1,t}\\ y_{A2,t}\\ y_{A3,t}\\ y_{B1,t}\\ y_{B2,t} \end{array}\right]=\left[\begin{array}{ccccc} 1 & 1 & 1 & 1 & 1\\ 1 & 1 & 1 & 0 & 0\\ 0 & 0 & 0 & 1 & 1\\ \; & \; & I_{5} & \; \end{array}\right]X\left[\begin{array}{c} y_{A1}\\ y_{A2}\\ y_{A3}\\ y_{B1}\\ y_{B2} \end{array}\right]$$

Compared to the original TSF problem, both forecasting accuracy of every series and aggregation consistency between levels are required in hierarchical forecasting. Aggregation consistency requires that the forecasts summation of the most disaggregated series are equal to the forecasts of the aggregated series; a constraint is known as coherence [3,5]. To ensure coherent forecasts, a two-step procedure is usually adopted in the literature. First, all of the time series at each level of the hierarchy are forecasted independently. These forecasts in this step are usually called “base” forecasts; however, they are usually not aggregated consistently. Therefore, it is often necessary to carry out a reconciliation approach in the second step to adjust the lower level forecasts and to make them coherent in the higher levels [5]. Many reconciliation approaches are introduced in the literature, including the bottom-up, top-down, and middle-out procedures [6,29].

It is demonstrated that the overall performance of these reconciliation approaches is not stable, and producing coherent forecasts is highly dependent on the application at hand. As an alternative, the optimal reconciliation approach using a linear regression algorithm is presented to overcome the limitations of the reconciliation approaches mentioned above, trying to produce more coherent forecasts [5,6,9]. Recently, different machine learning algorithms [12,14,15,16] are used to estimate the proportions of time series through the hierarchy as well as to derive the combination weights for the forecasts in the optimal combination approach. This paper develops a deep neural network-based transfer learning approach that runs in one-step, overcomes the limitations of state-of-the-art approaches, shows better prediction accuracy, and maintains coherency.

## 3. The Proposed Approach

In this section, we present a novel forecasting approach that exploits the potential of our previous model DLSTM [17] in auto-encoder fashion. In the following subsections, we provide more details about the elements of the proposed approach.

### 3.1. Transfer Learning in TSF

Recently, machine learning algorithms have been used widely to model and analyze different time series problems, including univariate, multivariate, and hierarchical architectures. In this concern, traditional machine learning algorithms, including ANNs, require the training and testing data to be sampled from the same domain, therefore having the same data distribution to perform a specific task. Nevertheless, we know that training ANNs require expensive resources and high computational costs, mainly when a similar process is performed for different tasks. The computational cost worsens and increases exponentially when the training model uses the DNN algorithm as a learning model. Transfer learning can address this problem and decrease the computation cost drastically.

**Figure 2:**
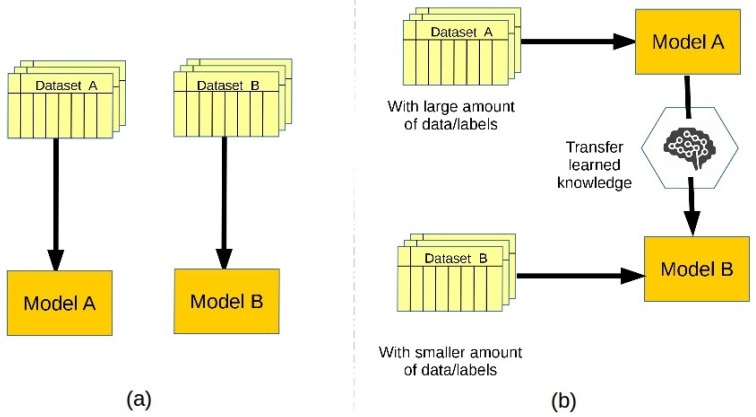
Representation of (**a**) traditional machine learning (**b**) transfer Learning.

In the general transfer learning approach, we first train a “base” model and learn meaningful features of a large-scope dataset to achieve a specific task. Then, we reuse the learned features, i.e., transfer them, onto another “target” network to be trained on a target dataset to perform a new, or similar, task [30]. In this way, transfer learning can overcome the limitations of traditional machine learning algorithms for training multiple deep learning models. Figure 2 simplifies a comparison between traditional machine learning and transfer learning algorithms. The traditional machine learning algorithms (Figure 2a) learn from a single dataset, and each traditional machine learning model operates separately. While transfer learning algorithms (Figure 2b) utilizes the knowledge gained from many source datasets, from a specific domain, and transfer them into the target domain. In this way, transfer learning significantly reduces the computational cost along with enhances the overall performance.

In our treatment of hierarchical forecasting in this paper, transfer learning is used to train the most disaggregated series, i.e. the bottom level, to produce the “base” forecasts. Then, leveraging the learned features in the bottom level to estimate the forecasts at the upper, or target, levels. Estimating the upper layers’ forecasts is executed in parallel, which reduces the computation costs, particularly in large hierarchies. In addition, it reduces the amount of data required for training the models through the hierarchy levels. Previously, transfer learning has applied for regression and classification problems in machine learning [31]. Recently, transfer learning have been applied for time series forecasting for real-world problems [32,33,34]. However, according to the best of our knowledge, this is the first time that transfer learning is employed in hierarchical forecasting problems.

### 3.2. Long Short-Term Memory (LSTM)

Sequence-to-sequence (seq2seq) learning is used in language translation, speech recognition, and recently in time series forecasting [35]. Conventional neural networks assumed that all observations of the input vectors are independent of each other. As a result, the traditional neural network cannot utilize the sequential information included in time series data. Contrary to the conventional neural networks, the recurrent neural network (RNN) approaches are used to generate a sequence of data such that each observation is supposed to be dependent on the previous ones [36]. As an elegant variation of RNN, LSTM is a recurrent neural network approach that can be applied to model sequential data as well [21]. RNN and its variants are trained to map an input sequence into an output sequence, where the network’s delay recursion enables it to represent the dynamic performance of sequential systems [37,38].

**Figure 3:**
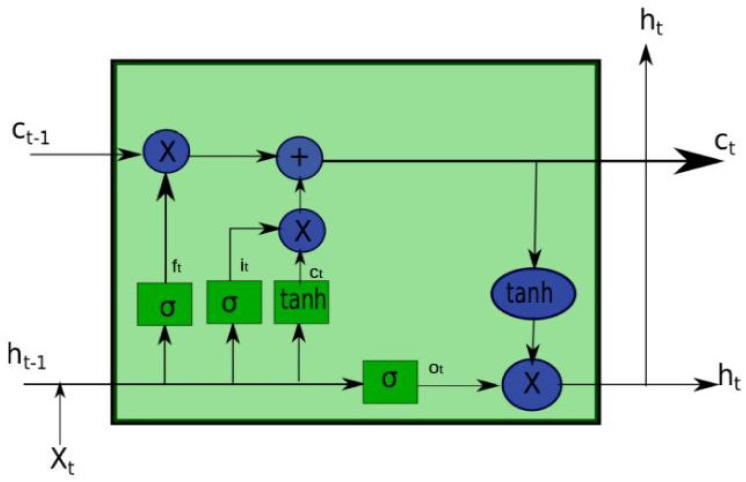
The internal structure of one LSTM cell.

As shown in Figure 3, the LSTM memory cell comprises four gates, namely, the input gate, the output gate, the forget gate, and the candidate (input modulation) gate; each has a different purpose. Precisely, the input gate controls whether to write data to the cell state. The output gate controls what data to pass as the output hidden state. The forget gate controls whether to erase data from the cell state. Finally, the candidate gate controls what data to write to the cell state. The following recursive equations show how the LSTM cell works.

(8) $$\begin{array}{ccc} i_{t} & = & \sigma (W_{xi}X_{t}+W_{hi}h_{t-1}+b_{i}) \end{array}$$

(9) $$\begin{array}{ccc} o_{t} & = & \sigma (W_{xo}X_{t}+W_{ho}h_{t-1}+b_{o}) \end{array}$$

(10) $$\begin{array}{ccc} f_{t} & = & \sigma (W_{xf}X_{t}+W_{hf}h_{t-1}+b_{f}) \end{array}$$

(11) $$\begin{array}{ccc} c_{t} & = & f_{t}⊙c_{t-1}+i_{t}⊙\tanh(W_{xc}X_{t}\\ & + & W_{hc}h_{t-1}+b_{c}) \end{array}$$

(12) $$\begin{array}{ccc} h_{t} & = & o_{t}⊙\tanh\left(c_{t}\right) \end{array}$$

where $i_{t}$, $o_{t}$, and $f_{t}$ are the LSTM gating for the cell state to input, output, and forget information. $c_{t}$ and $h_{t}$ are the cell memory state vector and the hidden state vector, respectively. σ represents the sigmoid function, and $x_{t}$ is the input vector. *Ws* are linear transformation matrices whose parameters need to be learned for each gate and cell memory, while *bs* is the corresponding bias vector. For simplicity, *LSTM* could be represented in one form as follows:

(13) $$h_{t},c_{t}=LSTM\left(x_{t},h_{t-1},c_{t-1}\right)$$

Recently, the first author of this paper introduced a deep LSTM (DLSTM) approach [17] as an extension of the shallow LSTM and used it to model various time series problems. The presented model includes a stack of LSTM blocks (or layers), one after another and interconnected in a deep architecture to combine the advantages of every single LSTM [39]. It runs as follows, each LSTM block operates at a different time scale and, therefore, processes a specific part of the desired task and, subsequently, passes it onto the following block until finally the last block that generates the network’s output [17].

The goal of stacking multiple LSTM blocks in such a hierarchical architecture is to build the features at the lower levels that disentangles the factors of variations in the input data and then combines these representations at the higher levels. In [17], we empirically showed that this deep architecture ensures the recovery of the limitations of shallow neural network architectures, particularly when long interval time series datasets are used. In addition, the empirical assessment showed that the DLSTM could efficiently represent the nonlinear relationship between the system inputs and outputs. These advantages can meet the requirements of generating stable and dynamic “base” forecasts, where the learned knowledge, or features, are transferred to the upper layers to obtain coherent forecasts.

### 3.3. Auto-Encoder (AE)

**Figure 4:**
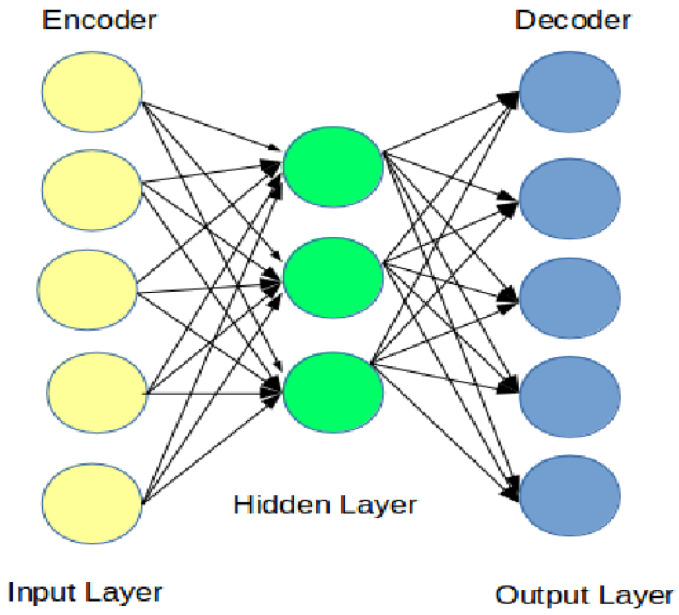
The auto-encoder architecture.

The AE is a neural network model often used for data generation. It consists of three layers, namely, the input layer (encoder), hidden layer, and output layer (decoder), as shown in Figure 4. The AE training procedure comprises two phases; encoding and decoding. In the encoding phase, the model learns a compressed representation (or latent variables) of the input. In contrast, in the decoding phase, the model reconstructs the target from the compressed representation during the encoding phase. The AE algorithm provides state-of-the-art results in seq2seq based applications, such as language translation, and sentiment analysis. Hence, the multi-input multi-output TSF problem can also be treated in a seq2seq fashion, as we will show in this paper.

The AE works by encoding the input *x*(*i*) and maps it into *h(x)* following to Equation (14). Next, the decoder phase maps *h* into the output *y*(*x*) following to Equation (15).

(14) $$\begin{array}{c} h\left(x\right)=f\left(W_{1}X+b_{1}\right) \end{array}$$

(15) $$\begin{array}{c} y\left(x\right)=g\left(W_{2}h+b_{2}\right) \end{array}$$

where $W_{1}$ and $b_{1}$ are the weight matrix the bias vector or the encoder, respectively. Whereas, the $W_{2}$ and $b_{2}$ are the same but for the decoder. The training of AE includes finding the parameters $W_{1},W_{2},b_{1},$ and $b_{2}$ that minimize the reconstruction error after back-propagating it through the network. The training objective is to minimize the reconstruction errors using the following squared errors, L=∥x−y∥.

### 3.4. DLSTM-Based Auto-Encoder

**Figure 5:**
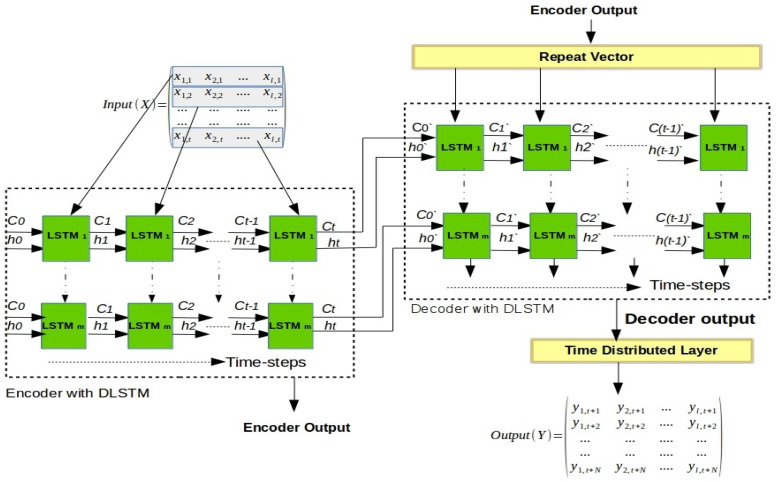
The DLSTM-based Auto-Encoder architecture.

The DLSTM-AE model is a novel architecture with the same structure as the original AE with some exceptions. As shown in Figure 5, we replace both the encoder layer with a DLSTM and the decoder layer with another DLSTM, which described in Section 3.2. The encoder layer converts the given input sequence into a fixed-length vector that acts as a summary of the input sequence. Usually, this fixed-length vector is called the context vector [40]. This context vector is fed as input to the decoder layer and the final encoder state as an initial decoder state to predict the output sequence. To use this architecture in a multi-step ahead forecasting, we add two layers, namely, repeat vector layer and time distributed dense layer. The repeat vector layer repeats the context vector, which we obtained from the encoder, and passes it as an input to the decoder. We repeat this for *n*-steps, where *n* is the number of future steps subject to forecast [41].

As the output received from the decoder concerning each time step is mixed, the time distributed densely applies a fully connected dense layer on each time step and separates the output for each time step [41]. Moreover, in the experiments of this paper, we used the proposed AE architecture in forecasting time series instead of reconstructing the input data. This deep architecture enables the model to learn complex and dynamic input sequential data from adjacent time intervals using multiple memory cells to remember long-term sequential data.

It is worth mentioning that the proposed DLSTM-AE is different from the LSTM-based Stacked Auto-Encoder (LSTM-SAE) model presented recently by Sagheer et al. [18]. In the LSTM-SAE, the AE uses two LSTMs, one as the encoder and the other as a decoder. Then, we built several models similar to this in a stacked fashion. However, the AE in the proposed approach maintains the original AE structure but with two DLSTMs: one DLSTM as an encoder and one DLSTM as a decoder, such that each DLSTM contains several hidden layers, typically as shown Section 3.2.

### 3.5. Methodology

The proposed methodology to manipulate the hierarchical forecasting uses the DLSTM-AE model and the transfer learning strategy in a straightforward and simple procedure. According to its properties, the DLSTM enables the proposed approach to extract complex features and to capture more information as long as the LSTM is going deeper in the bottom level [17]. Meanwhile, the generated forecasts in the upper levels are coherent and reconciled successfully by adopting the transfer learning strategy.

**Figure 6:**
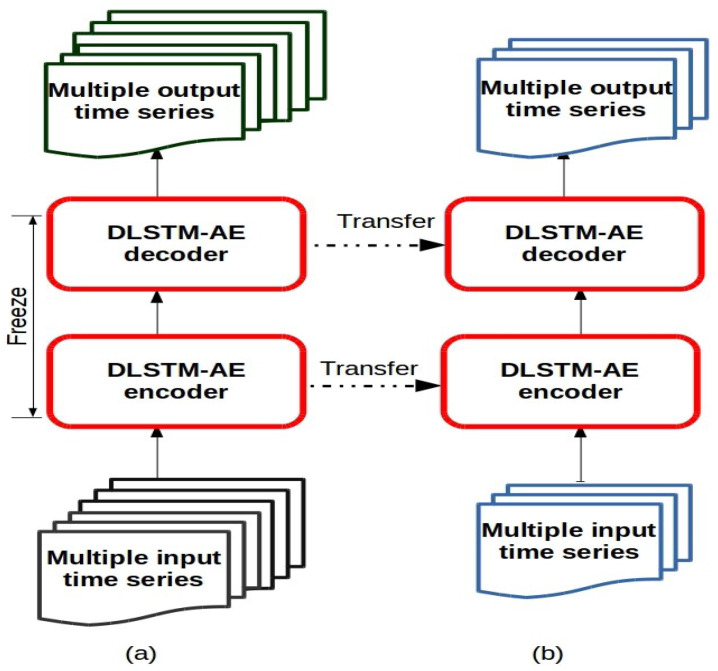
The DLSTM-AE model for (**a**) the bottom level (**b**) the upper levels.

As shown in Figure 6, the proposed methodology can be summarized in the following steps.

1. The encoder layer of the DLSTM-AE, which located at the bottom level of the hierarchy, receives as an input a sequence *X* of multiple time series that is treated jointly all at once as multivariate time series. This sequence can be represented as [*Xi*,*j*], where *i* = 1, …, *l* terms to time series in the *j* = 1, …, *t* time step, as shown in Figure 5.
2. The encoder DLSTM recursively handles the input sequence (*X*) of length *t* and updates the cell memory state vector $C_{t}$ and hidden state vector $h_{t}$ at each time step *t* using Equation (13). After (*t*) time steps, the encoder summarizes the whole input sequence into the final vectors $C_{t}$ and $h_{t}$.
3. A repeat vector layer takes the encoder output and passes it, as an input, to the decoder layer. Here, the function of the repeat vector is to repeat the decoder inputs to a number equals to future time steps that needed to perform the forecasting.
4. Then, the DLSTM at the decoder layer, at the bottom level, uses the final vectors $C_{t}$ and $h_{t}$ that passed from the encoder as initial cell memory state vector and initial hidden state vector $C_{0{}^{\prime }}$ and $h_{0{}^{\prime }}$ for $t{}^{\prime }$-length time step.
5. The decoder DLSTM learns the features that included in the original input data and yields multiple outputs with *N*-time step ahead in a single shot following the MIMO strategy that described in Section 2.2.
6. The time distributed densely applies a fully connected dense layer on each time step and separates the output for each time-step. In this step, we can evaluate the performance of the DLSTM-AE by estimating the prediction accuracy at the bottom level using different performance metrics.
7. Once the bottom level is trained, we maintain (or freeze) the neurons’ weight vectors of the DLSTM-AE model because they carry the features (or knowledge) of the input data. In the transfer learning terminology, this model is referred to as the “base” model.
8. We convey, or transfer, the trained DLSTM-AE model, or “base” forecast, to be used in the upper layers to learn the output layer of each upper level separately to generate the corresponding or “target” forecasts.

It is worth mentioning that taking the input (in steps 1–4) as a sequence of time series and producing multi-output in a single shot effectively reduces the overall computation. Additionally, based on the dataset and application in hands, we can implement the MIMO strategy (in step 5) either in one-step ahead or multi-step ahead prediction. We adopted nine-steps ahead for the experiments conducted in this paper, as shown in the empirical results section. As depicted in Figure 6, the trained models (in steps 1–6) are frozen before the transfer learning strategy that implemented in the higher levels in order to maintain, or not destroy, any of the information they contain [42].

As depicted in Figure 6, we utilize the “base” model to train the output layer in each higher level. At any higher level, it is possible that the length of the output sequences is smaller than the length of the input sequences included in the transferred “base” model. To solve this problem, zeros are inserted to each small-size sequence up to the established typical length in a process called zero-padding [43]. For auxiliary materials that may help to reproduce the proposed methodology, please refer to the GitHub page.

## 4. Experimental Setting

This section presents the details of datasets, performance metrics used, and implementation platforms.

### 4.1. Dataset-I: Brazilian Power Generation System

**Figure 7:**
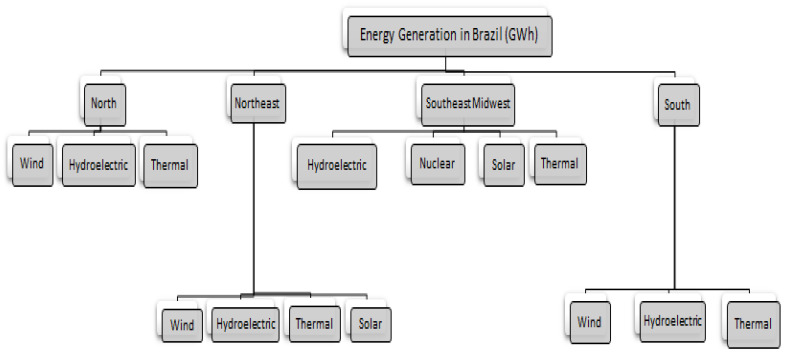
The Brazilian power generation system.

The first dataset utilized to assess the proposed approach corresponds to the amounts of power generation by each Brazilian electrical region, namely north, northeast, southeast/midwest, and south. Due to their reliability, the dataset was obtained from the Brazilian National Electric System Operator [44] as hourly power generation (*GWh*) between 1/2018 and 1/2020. The hierarchical aggregation structure of this dataset is demonstrated in Figure 7 and listed in Table 1. As we may notice, the structure of the dataset hierarchy allows us to model and analyze the hourly power generation in Brazil during the corresponding period.

In the experiment of this case study, we adhered to the experimental setting that adopted in other research papers that manipulate this dataset [19]. Specifically, the data are categorized based on source type of power energy (wind, hydroelectric, thermal, solar, and nuclear) for each region or subsystem. The observations of hourly power generation (in *GWh*) were collected from January 2018 to January 2020, making 17,521 *h*.

### 4.2. Dataset-II: Australian Domestic Tourism

In this experiment, we utilized the Australian domestic tourism dataset samples obtained from the National Visitor Survey, managed by TRA [45]. This dataset was collected annually by computer-assisted telephone interviews from more than 120,000 Australians aged 15 years. In this experiment, we followed the same procedure adopted in the literature that utilized the number of visitor nights as a sign of tourism activities. Then, we disaggregated the data based on a few purposes of travel: holiday, visiting friends and relatives, business, and others. The included observations span between 1998 to 2006, in a total of 36 quarterly observations. The structure of this hierarchy time series is displayed in Table 2.

In our experiments, we perform an out-of-sample forecast assessment to estimate all models using the first 12 observations (i.e., from 1998:Q1 to 2000:Q4) and then produced from 1-step ahead up to 8-step ahead forecasts. It is worth highlighting that we adhered to the experimental setting described in the original article to compare our proposed approach with it [20].

### 4.3. Performance Metrics

To ensure a fair assessment, several kinds of performance metrics are used widely in literature to calculate two main performance errors: scale-dependent errors and percentage-dependent errors. In all performance metrics, the error is defined as actual (or observed) value minus the forecasted value.

- Scale-dependent errors that are on the same scale as the data itself. The most common scale-dependent metric is the root mean square error (RMSE) [46], which calculates the square root of the mean of the squares of all errors. It can be given as follows:
  (16) $$RMSE=\sqrt{(}1/n\sum_{t=1}^{n}{\left(y_{t}-{\hat{y}}_{t}\right)}^{2})$$
  The RMSE is the most used metric in the prediction assessment; however, it can not ensure coherence between the observations and their prediction within the same series. Therefore, we enhance the assessment by calculating the dRMSE, which is the RMSE but applied for two consecutive prediction values. In other words, dRMSE calculates the difference, or variation, between two consecutive forecasts and their corresponding observations within the same series. Intuitively, the smaller the value of dRMSE, the better the performance is [47]. In this case, the dRMSE can be given as follows:
  (17) $$dRMSE=\sqrt{(}1/n\sum_{t=1}^{n}{\left(\delta y_{t}-\delta {\hat{y}}_{t}\right)}^{2})$$
- Percentage-dependent errors are more efficient in estimating the prediction precision since they have the advantage of being scale-independent. They are easy to understand because they provide the error in terms of percentages. Therefore, they are frequently used to assess the forecast performance among different scaled time series [17]. The most commonly used metric in this category is the mean absolute percentage error (MAPE), where percentage errors are summed regardless of the sign. MAPE can be given as follows:
  (18) $$\begin{array}{ccc} MAPE & = & 1/n\sum_{t=1}^{n}\left(|\left(y_{t}-{\hat{y}}_{t}\right)/y_{t}|\right)*100\;\left(\%\right) \end{array}$$

### 4.4. Hardware and Software Platforms

All experiments in this paper were implemented on an HP workstation-PC with an Intel Core™ i7-6700 CPU at 3.40 GHz, 8.00 GB RAM, ×64 based processor, equipped with an Ubuntu 16.04 operating system with python 3.7 software environment. The proposed approach was developed using the Keras library with an open-source TensorFlow [48] library in the back-end.

## 5. Empirical Results and Assessment

To assess the proposed approach, we investigated its performance in two case studies using the two datasets described in the previous section. In this assessment, we were concerned with checking two criteria: first, the forecasting accuracy and, second, the forecast coherency within the hierarchy. To demonstrate the efficiency, we compared the forecasting accuracy of the proposed approach with other reference methods in solving the same forecasting problems. It is worth highlighting that all results demonstrated in this section are based on the testing (or unseen) data in each case study. The last column in each table labeled “Average” shows the average values across all the forecast horizons for each approach.

### 5.1. Case Study I: Brazilian Electrical Power Generation

As depicted in Figure 7, this hierarchical dataset contains three levels. Therefore, the results displayed in Table 3 were estimated considering the hierarchical structure for the following levels,

- Level 0 (top): total power generation in Brazil
- Level 1 (middle): total power generation by four electrical subsystems (regions).
- Level 2 (bottom): total power generation by different fourteen sources.

For the first assessment criterion, which is related to forecasting accuracy, Table 3 shows the MAPE results of the proposed approach and other reference approaches. The tabulated results are computed from single-step up to nine-step ahead for all levels, where the average values are calculated and included in the last column in the table. The reference approaches are (BU) Bottom-up; (TDGSA) Top-down Gross-Sohl method A; (TDGSF) Top-down Gross-Sohl method F; (TDFP) Top-down forecast proportions; (OLS) Ordinary least-square; WLSs, Weighted least squares (structure scaling); WLSv, Weighted least squares (variance scaling); MinT (Sample), Minimum trace reconciliation; and MinT (Shrink), Minimum trace reconciliation (for more information about these models, please refer to [19]). All of the reference approaches generated the “base” forecasts using the ARIMA method, whereas the proposed approach generated the “base” forecast using the DLSTM-AE model.

**Figure 8:**
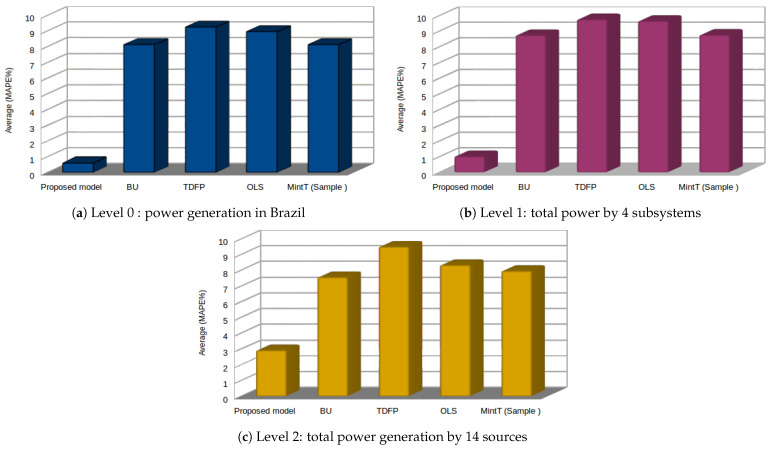
Performance comparison based on the MAPE metric for each level in case study I between the proposed approach and reference approaches.

It is easy to notice that the proposed approach attains a minor percentage error than other reference approaches presented in the table. Regarding the reference approaches, which are ARIMA-based, the results obtained using the top-down (TD) procedures show the highest percentage errors in all levels. Both the bottom-up (BU) and top-down (TD) approaches have another drawback; namely, they do not consider the correlation among the time series at each level. The MinT (Sample) approach attains the second-best performance after our approach, particularly for the one-step ahead forecast, among the ARIMA-based approaches. The MAPE values tend to be stable for the last three hours of the forecasting horizon. Figure 8 displays visual representations of the comparison between the proposed and reference approaches based on the average values.

Besides the MAPE performance metric, we performed other assessments using the RMSE and dRMSE metrics. Table 4 shows the results of these two metrics for the proposed approach only because the authors in [19] did not used these two metrics. The RMSE values are the difference between the predicted value and the corresponding observation for each time series on the same level. We can notice the small values of this metric, particularly in the earlier horizons or low-step ahead.

As a special case from the RMSE, the dRMSE metric calculates the difference between two consecutive predictions and their corresponding values. In this way, we can ensure that the dRMSE refers to the accuracy of the predicted variations. Accordingly, this performance metric identifies the relationship and inherent correlation included in a time series. From Table 4, it is clear that the results values range from 0.2 and 0.4 for the top level and range from 1.7 and 9.3 for the middle level. However, the error values tend to decrease as the horizon increases. Moreover, dRMSE attains minor error values at the top level of the hierarchy compared to the lower level. This means that, at the higher levels of hierarchy, the forecast values tend to be more correlated. Overall, the dRMSE values are not optimum to some extent; a phenomenon may be attributed to the diversity or divergence that exists among the original data [19]. We attributed this attitude to the diversity of original data because we do not note this phenomenon in case study II.

The second criterion we consider in our assessment is the ability to produce coherent forecasts more simply than that adopted in reference approaches. Table 5 shows the values of the MSE performance metric for each level, after the bottom-level. The computations are performed in two steps: First, the forecasts for every series at the bottom level were summed to generate forecasts for all higher levels in the hierarchy. Second, we calculated the difference between these forecast values and the corresponding forecast values produced by the proposed approach for the same level. Intuitively, as long as this difference is tiny, the proposed approach yields coherent forecasts. It is easy to notice that most differences in all horizons are very tiny, particularly as we are going deeper in the hierarchy. This indicates that the proposed approach maintains forecast coherency through the hierarchy.

### 5.2. Case Study II: Australian Visitor Nights of Domestic Tourism

This hierarchical dataset contains four levels, therefore, the results displayed in Table 6 were estimated considering the hierarchical structure for the following levels:

- Level 0 (top): total visitors in Australia;
- Level 1 (middle): total visitors according to the purpose of travel, with total four travel purposes;
- Level 2 (middle): total visitors according to a state visit, with a total of 28 state visits;
- Level 3 (bottom): total visitors according to city visit, with a total of 56 visits.

For the first assessment of the forecasting accuracy, Table 6 shows the MAPE measurements of the proposed approach and other reference models. The results are shown for all levels from a single-step up to eight-steps ahead, where the average values are calculated and included in the last column of the table. The reference models in this case study are bottom-up, top-down based on historical proportion (HP1 and HP2), top-down based on forecast p (FP), and optimal combination (for more information about the setting of these models, please refer to [20]). All the reference models generated the “base” forecast using the exponential smoothing statistical technique, whereas the proposed approach generated the “base” forecast using the proposed DLSTM-AE model.

**Figure 9:**
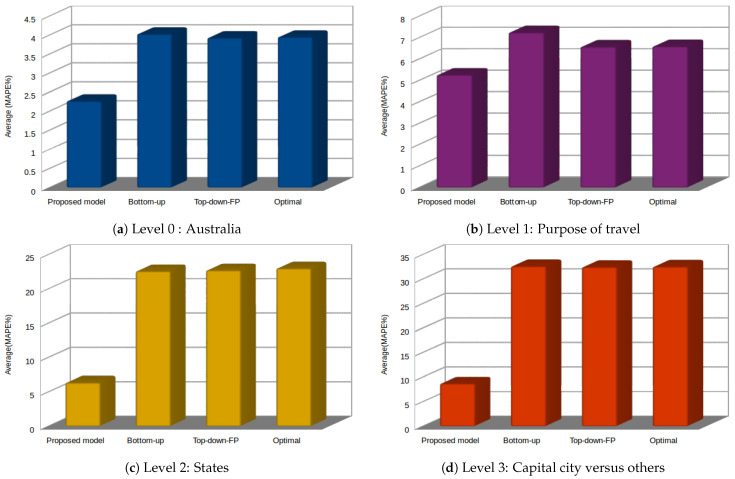
Average performance comparison for each level in Case Study II: Australian visitor nights of domestic tourism.

In Table 6, we can easily notice that the proposed approach attains a minor percentage error compared to other reference approaches displayed in the table. Regarding the reference models, the results obtained using the top-down procedure based on historical proportions (top-down HP1 and top-down HP2) show the highest percentage errors in all the hierarchy levels; however, they attain good results for forecasting at the top level of the hierarchy. The top-down procedure based on historical proportions (top-down FP) and optimal combination procedure attain the second-best performances after our proposed approach. In contrast, the bottom-up procedure performs better at the two top levels of the hierarchy and the worst at the two bottom levels. Figure 9 displays visual representations of this comparison, where we can notice the superiority of the proposed approach’s performance, particularly at the bottom level and the two middle levels.

Besides the MAPE performance metric, we conducted another assessment using the RMSE and dRMSE performance metrics. Table 7 shows the results of these two metrics for the proposed approach only because the authors in [20] did not consider these two metrics. Again, the RMSE value is the difference between the predicted value and the corresponding observation for each time series on the same level. We can notice the tiny values of this metric attained by the approach, particularly in the top layers of the hierarchy.

As defined before, the dRMSE metric calculates the difference between two consecutive predictions and their corresponding values. By this metric, we can identify the relationship and inherent correlation that exist in a time series. In Table 7, it is easy to notice that the error values tend to decrease as the horizon increases for all levels. As in case study I, the dRMSE showed more minimal values at the top level of the hierarchy than the lower levels. Again, this means that the forecast values tend to be more correlated at higher hierarchy levels. In contrast to case study I, the dRMSE values here are still ideal and attained lower values, which confirm our conclusion that the high values of dRMSE in case study I may be attributed to the heterogeneity of the original data samples.

In this case study, we continue to investigate the proposed approach’s capability to produce coherent forecasts in a straightforward way compared to that utilized in other approaches. Table 8 shows the values of the MSE performance metric for each level in the same way that described in case study I. As we explained in the previous case study, as long as this difference is very small, the proposed approach produces coherent forecasts. It is clear that most differences in all horizons are very tiny, particularly for earlier horizons or low-step ahead. Newly, this confirms that the proposed approach maintains forecast coherency for all levels in the hierarchy.

## 6. Overall Discussion and Conclusions

Forecasting through hierarchical time series has always been a challenging task for traditional approaches and various machine learning approaches. The challenge here is twofold; the first is to attain high prediction accuracy through the hierarchical architecture. The second is to ensure the forecasting consistency among hierarchy levels based on their dimensional features, a phenomenon called coherency. However, existing approaches exhibit good results addressing these two challenges, often showing instability in the overall prediction performance. This is because these approaches aim to minimize the prediction errors and to obtain good accuracy without questioning the coherence of consecutive predictions. As the granularity at which forecasts are needed increases, existing approaches may not scale well.

This paper addressed the hierarchical time series forecasting problem by extending our recent achievement, namely, the DLSTM model. Here, we reproduced the DLSTM model in auto-encoder (DLSTM-AE) fashion and implemented it in a transfer learning scenario. The proposed approach runs as follows: we first train the time series of the bottom level using DLSTM-AE to generate the “base” forecasts. Then, we freeze the weight vector of the “base” models and transferred the learned features to achieve synchronous training to the time series of the upper levels in the hierarchy.

Toward a fair evaluation, we compared the performance of the proposed approach with several existing approaches using two case studies belonging to different domains. The evaluation was based on two criteria: forecasting accuracy and the ability to produce coherent forecasts. The performance of all contenders was evaluated using three different performance metrics through multi-step ahead prediction mode. In both case studies, the proposed approach attained the highest accuracy results compared to other counterparts. We can attribute the goodness of the proposed approach to the use of DLSTM in generating the “base” forecasts at the bottom level compared to the existing approaches that use traditional statistical techniques, such as ARIMA and ES, to generate it. In this way, the individual forecasts at the bottom level are utilized on higher hierarchy levels to rapidly generate global forecasts without conducting a time-consuming parameter estimation as in existing approaches.

Moreover, the DLSTM-AE considered the relationship and temporal correlations among time series data and automatically extracted many inherent useful features that helped to generate the “target” forecasts. In contrast, most traditional techniques are static and ignore more helpful information. With the help of transfer learning, we significantly reduced the time for calculating the forecasts and substantially increase the forecasting efficiency for the higher level entities. The superiority of the proposed approach was not noticed only for the forecasting accuracy but also for the production of coherent forecasts. Using a standard performance metric, we empirically found that the difference between the forecast of a time series at a higher level and the sum of the corresponding time series forecasts at a lower level is tiny. This means that the proposed approach generated more coherent and consistent forecasts compared to reference models.

For future work, we plan to ensure forecast coherency at all levels of the hierarchy by running the proposed approach in a cross-temporal framework. Besides coherency, this framework will reduce the effect of the outliers and enhance the signal-to-noise ratio at aggregated lower frequencies of the time series while mitigating loss of information. In addition, despite describing our approach exemplarily through the tourism and energy domains, it can be easily adapted to other domains such as supply chain, sales promotion, and retail. 

## Figures and Tables

**Table 1:** Dataset-I: Brazilian power generation hierarchy data structure.

Level (No.)	Group	No. of Series Per Level
(0)	Power energy generation system in Brazil	1
(1)	Power energy generation subsystems (regions)	4
(2)	Power energy generation sources	14

**Table 2:** Dataset-II: Australian domestic tourism hierarchy data structure.

Level (No.)	Group	No. of Series Per Level
(0)	Australia	1
(1)	Purpose of Travel	4
(2)	States and Territories	28
(3)	Capital city versus others	56

**Table 3:** Performance comparison based on the MAPE performance metric between the proposed approach and the reference approaches to solve the Brazilian power generation problem as reported in [19].

MAPE	Forecast Horizon (*h*)									
	1	2	3	4	5	6	7	8	9	Average
	Level 0 (Top): Total Electrical Power in Brazil									
**Proposed**	**0.04**	**0.87**	**0.80**	**0.85**	**0.83**	**0.83**	**0.76**	**0.74**	**0.48**	**0.60**
BU [19]	2.00	3.53	5.62	8.04	10.17	11.45	11.17	10.76	10.48	8.14
TDGSA [19]	2.07	3.76	6.10	8.79	11.25	12.87	12.95	12.72	12.63	9.24
TDGSF [19]	2.07	3.76	6.10	8.79	11.25	12.87	12.95	12.72	12.63	9.24
TDFP [19]	2.07	3.76	6.10	8.79	11.25	12.87	12.95	12.72	12.63	9.24
OLS [19]	1.98	3.65	5.94	8.59	10.99	12.54	12.55	12.23	12.06	8.95
WLSv [19]	1.91	3.51	5.70	8.24	10.51	11.93	11.79	11.32	10.99	8.43
WLSs [19]	1.88	3.46	5.63	8.15	10.39	11.77	11.59	11.09	10.76	8.30
MintT (Sample ) [19]	1.68	3.29	5.50	8.02	10.24	11.57	11.31	10.85	10.60	8.12
MinT (Shrink) [19]	1.74	3.36	5.59	8.12	10.35	11.69	11.44	10.94	10.69	8.21
	Level 1—Electrical Subsystems									
**Proposed**	**0.17**	**0.98**	**1.2**	**1.31**	**1.21**	**1.16**	**1.09**	**1.06**	**1.06**	**1.02**
BU [19]	1.97	3.64	6.12	8.75	10.78	11.93	11.90	11.70	11.88	8.74
TDGSA [19]	31.97	31.74	30.37	28.93	28.12	27.49	26.71	26.04	25.36	28.53
TDGSF [19]	32.38	32.14	30.71	29.21	28.21	27.46	26.71	26.06	25.41	28.70
TDFP [19]	1.86	3.88	6.68	9.89	9.89	12.52	14.19	14.45	14.34	9.75
OLS [19]	1.90	3.55	6.30	9.20	11.70	13.36	13.64	13.56	13.66	9.65
WLSv [19]	1.77	3.35	5.84	8.62	10.84	12.38	12.56	12.40	12.41	8.91
WLSs [19]	1.81	3.41	5.92	8.74	11.00	12.57	12.79	12.68	12.75	9.07
MintT (Sample ) [19]	1.64	3.20	5.66	8.50	10.76	12.23	12.40	12.21	12.20	8.76
MinT (Shrink) [19]	1.66	3.28	5.75	8.57	10.84	12.33	12.50	12.31	12.28	8.83
	Level 2—Sources									
**Proposed**	**0.60**	**1.20**	**3.89**	**4.26**	**3.76**	**3.42**	**3.01**	**3.01**	**3.02**	**2.91**
BU [19]	2.66	5.05	6.53	7.71	8.88	9.46	9.40	9.22	9.11	7.56
TDGSA [19]	46.33	44.34	41.72	40.35	39.87	39.29	38.44	37.52	36.58	40.49
TDGSF [19]	47.66	45.70	42.87	41.24	40.42	39.64	38.80	37.90	36.96	41.24
TDFP [19]	2.83	5.51	7.53	9.45	9.45	11.46	12.79	13.20	13.33	9.50
OLS	2.51	5.07	6.78	8.29	9.78	10.62	10.73	10.63	10.56	8.33
WLSv [19]	2.60	5.11	6.74	8.09	9.42	10.18	10.21	10.07	9.97	8.04
WLSs [19]	2.56	4.98	6.64	8.00	9.31	10.00	9.95	9.70	9.63	7.86
MintT (Sample ) [19]	2.48	4.96	6.58	7.91	9.27	10.10	10.22	10.10	10.00	7.96
MinT (Shrink) [19]	2.52	5.04	6.68	8.02	9.38	10.20	10.30	10.19	10.10	8.05

**Table 4:** Hierarchical forecasting for Brazilian power generation using other performance metrics.

Metric	Forecast Horizon (*h*)								
	**1**	**2**	**3**	**4**	**5**	**6**	**7**	**8**	**9**
	Level 0: Total—Brazil								
RMSE	5.2 × 10${}^{-4}$	8.8 × 10${}^{-3}$	9.2 × 10${}^{-3}$	9.8 × 10${}^{-3}$	9.9 × 10${}^{-3}$	9.7 × 10${}^{-3}$	9.2 × 10${}^{-3}$	8.2 × 10${}^{-5}$	8.04 × 10${}^{-3}$
dRMSE	0.299	0.312	0.35	0.33	0.35	0.42	0.43	0.45	0.24
	Level 1: Electrical subsystems								
RMSE	2.6 × 10${}^{-3}$	0.012	0.014	0.014	0.014	0.014	0.014	0.014	0.02
dRMSE	9.3	6.68	6.71	5.67	5.81	6.30	6.53	7.50	1.68

**Table 5:** Hierarchical forecasting for Brazilian power generation: Coherence performance metric.

Metric	Forecast Horizon (*h*)								
	**1**	**2**	**3**	**4**	**5**	**6**	**7**	**8**	**9**
	Level 0: Total—Brazil								
MSE	1.15 × 10${}^{-6}$	0.001	0.002	0.003	0.005	0.006	0.008	0.009	0.09
	Level 1: Electrical subsystems								
MSE	4.2 × 10${}^{-6}$	0.003	0.0003	0.0003	0.0002	0.0002	0.0001	0.0001	0.0002

**Table 6:** Performance comparison based on the MAPE performance metric between the proposed approach and the reference approaches to solve the Australian tourism visitor nights problem as reported in [20].

MAPE	Forecast Horizon (h)								
	**1**	**2**	**3**	**4**	**5**	**6**	**7**	**8**	**Average**
Level 0: Australia									
**Proposed**	**3.17**	**1.12**	**1.55**	**1.90**	**2.41**	**2.47**	**2.89**	**2.61**	**2.27**
Bottomup [20]	3.48	3.30	3.81	4.04	3.90	4.56	4.53	4.58	4.03
Top-down HP1 [20]	3.89	3.71	3.41	3.90	3.91	4.12	4.27	4.27	3.93
Top-down HP2 [20]	3.89	3.71	3.41	3.90	3.91	4.12	4.27	4.27	3.93
Top-down FP [20]	3.89	3.71	3.41	3.90	3.91	4.12	4.27	4.27	3.93
Optimal [20]	3.80	3.64	3.48	3.94	3.85	4.22	4.34	4.35	3.95
Level 1: Purpose of travel									
**Proposed**	**4.98**	**4.51**	**4.57**	**3.65**	**3.38**	**3.50**	**4.51**	**5.00**	**5.26**
Bottom-up [20]	6.15	6.22	6.49	6.99	7.80	8.15	8.21	7.88	7.24
Top-down HP1 [20]	9.83	9.34	9.34	9.67	9.81	9.52	9.88	9.81	9.65
Top-down HP2 [20]	10.01	9.56	9.55	9.84	9.98	9.71	10.06	9.97	9.84
Top-down FP [20]	5.73	5.78	5.58	6.15	6.80	7.28	7.56	7.68	6.57
Optimal [20]	5.63	5.71	5.74	6.14	6.91	7.35	7.57	7.64	6.59
Level 2: States									
**Proposed**	**8.47**	**7.98**	**6.54**	**6.58**	**6.02**	**5.14**	**4.91**	**5.07**	**6.34**
Bottom-up [20]	21.34	21.75	21.81	22.39	23.76	23.26	23.01	23.31	22.58
Top-down HP1 [20]	32.63	30.98	31.49	31.91	32.23	30.11	30.51	30.91	31.35
Top-down HP2 [20]	32.92	31.23	31.72	32.13	32.47	30.32	30.67	31.01	31.56
Top-down FP [20]	22.15	21.96	21.94	22.52	23.79	23.18	22.96	23.07	22.70
Optimal [20]	22.17	21.80	22.33	23.53	24.26	23.15	22.76	23.90	22.99
Level 3: Capital city versus other									
**Proposed**	**9.51**	**9.00**	**7.71**	**8.70**	**8.51**	**7.42**	**6.91**	**6.82**	**8.07**
Bottom-up [20]	31.97	31.65	31.39	32.19	33.93	33.70	32.67	33.47	32.62
Top-down HP1 [20]	42.47	40.19	40.57	41.12	41.71	39.67	39.87	40.68	40.79
Top-down HP2 [20]	43.04	40.54	40.87	41.44	42.06	39.99	40.21	40.99	41.14
Top-down FP [20]	32.16	31.30	31.24	32.18	34.00	33.25	32.42	33.22	32.47
Optimal [20]	32.31	30.92	30.87	32.41	33.92	33.35	32.47	34.13	32.55

**Table 7:** Hierarchical forecasting for Australian tourism visitor nights using other performance metrics.

Measure	Forecast Horizon (*h*)							
	1	2	3	4	5	6	7	8
Hierarchical Level 0: Total—Australia								
RMSE	0.03	0.014	0.014	0.014	0.02	0.03	0.03	0.03
dRMSE	0.69	0.67	0.45	0.52	0.37	0.37	0.33	0.05
Hierarchical level 1: Purpose of travel								
RMSE	0.05	0.05	0.04	0.04	0.04	0.04	0.05	0.07
dRMSE	1.05	0.82	0.59	0.54	0.49	0.40	0.28	0.12
Hierarchical level 2: States								
RMSE	0.14	0.14	0.12	0.14	0.13	0.13	0.13	0.13
dRMSE	0.53	0.27	0.26	0.18	0.17	0.16	0.14	0.19

**Table 8:** Hierarchical forecasting for Australian tourism visitor nights: Coherence performance metric.

Metric	Forecast Horizon (*h*)							
	1	2	3	4	5	6	7	8
	Hierarchical level 0: Total—Australia							
MSE	0.03	0.016	0.015	0.02	0.023	0.021	0.021	0.025
	Hierarchical level 1: Purpose of travel							
MSE	0.0009	0.001	0.0019	0.0014	0.0011	0.0011	0.0019	0.0026
	Hierarchical level 2: States							
MSE	0.00061	0.0008	0.9	0.1	0.1	0.03	0.05	0.1

## Data Availability

The dataset of Australian tourism visitor nights is publicly available at: https://robjhyndman.com/data/hier1_with_names.csv accessed on 25 June 2021.
